# Unusual Transmission of *Plasmodium falciparum*, Bordeaux, France, 2009

**DOI:** 10.3201/eid1702.100595

**Published:** 2011-02

**Authors:** Marc-Olivier Vareil, Olivier Tandonnet, Audrey Chemoul, Hervé Bogreau, Mélanie Saint-Léger, Maguy Micheau, Pascal Millet, Jean-Louis Koeck, Alexandre Boyer, Christophe Rogier, Denis Malvy

**Affiliations:** Author affiliations: Centre Hospitalier Universitaire Pellegrin, Bordeaux, France (M.-O. Vareil, M. Saint-Léger, A. Boyer);; Hôpital d’Enfants, Bordeaux (O. Tandonnet, M. Micheau); Hôpital d’Instruction des Armées, Bordeaux (A. Chemoul, J.-L. Koeck);; Institut de Médecine Tropicale du Service de Santé des Armées, Marseille, France (H. Bogreau, C. Rogier);; Université Victor Segalen, Bordeaux (P. Millet, D. Malvy);; Centre Hospitalier St-André, Bordeaux (D. Malvy)

**Keywords:** *Plasmodium falciparum*, newborn, transfusion, professional blood exposure, parasites, dispatch

## Abstract

*Plasmodium falciparum* malaria is usually transmitted by mosquitoes. We report 2 cases in France transmitted by other modes: occupational blood exposure and blood transfusion. Even where malaria is not endemic, it should be considered as a cause of unexplained acute fever.

Unusual forms of parasitic infection, such as those acquired by blood transfusion (*1*,*2*) or accidental exposure to infected blood (*3*), may be challenging to diagnose in areas where these infections are not endemic (*4*). We report 2 cases of *Plasmodium falciparum* malaria transmitted by routes other than mosquito vectors: occupational blood exposure and blood transfusion.

## The Patients

Patient 1, a 36-year-old woman who worked as a technician in a clinical laboratory, was admitted to Bordeaux Hospital, France, on June 2, 2009. She had a 7-day history of high fever (up to 39°5 C), rigors, headache, and jaundice. Hematologic tests showed decreased platelets (33,000 × 10^9^ platelets/L) and increased serum C-reactive protein (130 mg/L; reference <5 mg/L). Although the patient denied having traveled abroad in the past 5 years, she lived near an international airport, and her clinical signs were typical of malaria. Consequently, thick and thin blood smears were performed and indicated *P. falciparum* parasitemia of 0.1%. Serum bilirubin was 86 μmol/L (reference 3–18 μmol/L). By 6 hours after hospital admission, her condition had dramatically worsened, with hemodynamic collapse (blood pressure 60/32 mm Hg) associated with macroscopic hemoglobinuria; parasitemia increased to 6%. All viral and bacteriologic testing results were negative. Treatment was intravenous quinine formate (loading dose 17 mg/kg, followed by maintenance dose of 8.3 mg/kg 3×/d). After 5 days, her medication was switched to oral quinine for another 2 days. Parasitemia was absent 6 days after starting quinine, and the patient was discharged 2 days later. Blood smears were negative 21 days after discharge.

Patient 1 later recalled that 2 weeks before hospital admission she had been injured by a broken, blood-contaminated, malaria diagnostic (QBC) test tube at work. Because she considered the incident trivial, she did not inform her workplace of it. Had she done so, the standard blood exposure protocol would have been automatically triggered. The source of the blood was subsequently traced to a patient returning from the Congo, for whom *P. falciparum* parasitemia of 4% had been diagnosed when the blood sample was taken. Genotyping of blood samples from patient 1 and the presumed source traveler were performed. *P. falciparum* isolates were genotyped at the 7A11, C4M79, Pf2802, and Pf2689 microsatellite loci and at the highly polymorphic loci of the merozoite surface protein 1 and 2 antigen genes by fluorescent end-labeled nested PCR and restriction fragment length polymorphism analysis (*5*–*7*). Results showed that the 2 infections had the same molecular signature and complete homology and were confirmed by genotypic analysis of resistance markers. A threonine variant on codon K76T point mutation of *P. falciparum* chloroquine resistance transporter and an identical level of resistance to antifolate drugs (*P. falciparum* dihydrofolate reductase–thymidylate synthase point mutations at positions 51, 59, and 108) were found (Table) (*5*,*7*). A diagnosis of severe malaria as a result of occupational percutaneous blood exposure was therefore retained.

**Table:** Genotyping results for 2 *Plasmodium falciparum* isolates, France, 2009*

Locus	Secondary case			Index case	
Type	Allele	Type	Allele
Microsatellites†					
7A11	NA	119 bp		NA	119 bp
C4M79	NA	203 bp		NA	203 bp
Pf2802	NA	139 bp		NA	139 bp
Pf2689	NA	87 bp		NA	87 bp
Antigen genes‡					
* msp-1*	Ro33	149 bp		Ro33	149 bp
* msp-2*	FC27	413 bp		FC27	413 bp
Drug resistance genes§					
*pfcrt* codon 76	Mutated	ACA		Mutated	ACA
* pfdhfr*					
Codon 16	Wild-type	GCA		Wild-type	GCA
Codon 51	Mutated	ATT		Mutated	ATT
Codon 59	Mutated	CGT		Mutated	CGT
Codon 108	Mutated	AAC		Mutated	AAC
Codon 164	Wild-type	ATA		Wild-type	ATA

*NA, not applicable; *msp*, merozoite surface protein; *pfcrt*, *P. falciparum* chloroquine resistance transporter; *pfdhfr*, *P. falciparum* dihydrofolate reductase. †Size of amplified DNA fragment expressed. ‡Allelic family and size of amplified DNA fragment expressed. §Single-nucleotide polymorphism. *pfcrt* codon 76 point mutation was genotyped by nested PCR and restriction fragment-length polymorphism analysis. *pfdhfr* point mutations at positions 16, 51, 59, 108, and 164 were genotyped by nested PCR and primer expansion. Underlining indicates nucleotide positions that can be mutated.

Patient 2 was a 15-day-old girl who was referred from Dakar, Senegal, where she had been born on March 11, 2009. She was the third child of a Lebanese family who had no known genetic illness and who lived in an air-conditioned house. The pregnancy had been uneventful, and the infant was delivered at term. Her blood group was B Rh+. At day 14 after birth, pallor and jaundice suggested severe neonatal anemia. Blood testing indicated decreased hemoglobinemia (5.2 g/dL; reference 9–14 g/dL) and reticulocytosis (260,000 × 10^9^ cells/L; reference 25,000–85,000 × 10^9^ cells/L). Serum C-reactive protein was <5 mg/L. The patient received a 60-mL whole blood transfusion from a compatible donor in Dakar on March 26; the next day, she was transferred to Bordeaux Hospital.

At the time of admission, hematologic testing confirmed severe regenerative hemolytic anemia (hemoglobin 9.5 g/dL, reticulocytes 320,000 × 10^9^ cells/L, and serum haptoglobin <0.08 g/L [reference 0.80–2.15 g/L]). Abdominal and transfontanellar echography showed no abnormalities. PCR specific for parvovirus B19 and cytomegalovirus was negative. Serologic testing for malaria, thick and thin blood smear, and specific PCR for *P. falciparum pBRK1–14* in the newborn and her mother were negative. Investigation for ABO blood incompatibility or immunologically related hemolysis produced negative results. Screening of both parents and the newborn for inherited hemolysis excluded an erythrocyte membrane abnormality. Hemoglobinopathy and erythrocyte enzyme (glucose 6-phosphate dehydrogenase, pyruvate kinase, and hexokinase) deficiencies were also ruled out. The newborn and her parents were followed up weekly; no pertinent clinical findings or abnormal PCR and blood smear were found until 7 weeks after birth, when febrile thrombocytopenia (92,000 × 10^9^ platelets/L) and neutropenia (540 × 10^9^ cells/L) were detected.

At 7 weeks after birth, blood smears indicated *P. falciparum* parasitemia of 6%. Treatment with intravenous quinine formate at 8.3 mg/kg 3×/d for 4 days, followed by oral mefloquine (20 mg/kg) for 1 day led to prompt improvement. Follow-up at 3 and 5 months of age showed resolution of anemia and no relapse of malaria. Test results for hemolysis remained negative, and glucose 6-phosphate deshydrogenase and erythrocyte pyruvate kinase levels 6 months after transfusion were within normal limits. Although it could not be confirmed, neonatal anemia was attributed to fetal–maternal hemorrhage.

We contacted the clinic in Dakar, where the blood sample but not the donor could be traced. However, a thick and thin blood smear examination subsequently performed in Dakar was positive for *P. falciparum*, clearly supporting a diagnosis of transfusion-transmitted malaria.

## Conclusions

In non–malaria-endemic countries, accidental blood-borne inoculation of *P. falciparum* after direct occupational exposure of health care or laboratory personnel has been rarely reported (*8*–*10*). The course of such occupationally acquired malaria may be critical if the inoculating injury is neglected or unrecognized, as it was by patient 1. This case emphasizes the need for laboratories handling blood to ensure that every accidental injury, however trivial it may seem, is declared and managed. As occurred with patient 1, a delayed diagnosis may also delay delivery of appropriate care for preventing severe or complicated illness (*9*,*10*).

Whereas most non–malaria-endemic countries, including France, are implementing selective screening strategies for blood product donors (*11*), prevention of transfusion-transmitted malaria in endemic areas such as Senegal remains a challenge because such screening is not routinely performed. For patients such as patient 2, malaria is rarely diagnosed in non–malaria-endemic countries (*12*,*13*). For this patient, the chronology of events and exposure to blood from a contaminated donor are highly suggestive of transfusion-transmitted malaria. In the absence of immunologic indicators that the mother had been exposed to *P. falciparum*, vertical transmission is extremely unlikely, and the risk for mosquito transmission is low because of the family’s living environment and the time of year. Nevertheless, without gene sequencing of parasites from the donor, transfusion-transmitted malaria cannot be demonstrated unequivocally.

Neonates are assumed to be able to counteract natural infection with malaria because of the predominance of fetal hemoglobin, which is not suitable for complete erythrocyte schizogony of *P. falciparum* (*14*). For patient 2, transfusion with infected adult blood may have compromised this capacity, enabling the infection to follow its natural course. In Senegal, blood transfusion recipients are routinely given antimalaria treatment (*15*). Unfortunately, such treatment cannot be extended to neonates because of lack of a validated and convenient therapeutic antimalaria regimen for this age group.

The above-mentioned types of malaria transmission are unusual. However, in non–malaria-endemic countries, a recent history of blood transfusion or an episode of accidental inoculation of blood may account for malaria infection in persons who are not otherwise at risk.
